# Analytical derivation of elasticity in breast phantoms for deformation tracking

**DOI:** 10.1007/s11548-018-1803-x

**Published:** 2018-06-04

**Authors:** Vincent Groenhuis, Francesco Visentin, Françoise J. Siepel, Bogdan M. Maris, Diego Dall’alba, Paolo Fiorini, Stefano Stramigioli

**Affiliations:** 1 University of Twente, Drienerlolaan 5, 7522 NB Enschede, The Netherlands; 2 University of Verona, Strada le Grazie 15, 37134 Verona, Italy

**Keywords:** Biopsy, Magnetic resonance imaging, Elastic calibration, Breast

## Abstract

**Purpose:**

Patient-specific biomedical modeling of the breast is of interest for medical applications such as image registration, image guided procedures and the alignment for biopsy or surgery purposes. The computation of elastic properties is essential to simulate deformations in a realistic way. This study presents an innovative analytical method to compute the elastic modulus and evaluate the elasticity of a breast using magnetic resonance (MRI) images of breast phantoms.

**Methods:**

An analytical method for elasticity computation was developed and subsequently validated on a series of geometric shapes, and on four physical breast phantoms that are supported by a planar frame. This method can compute the elasticity of a shape directly from a set of MRI scans. For comparison, elasticity values were also computed numerically using two different simulation software packages.

**Results:**

Application of the different methods on the geometric shapes shows that the analytically derived elongation differs from simulated elongation by less than 9% for cylindrical shapes, and up to 18% for other shapes that are also substantially vertically supported by a planar base. For the four physical breast phantoms, the analytically derived elasticity differs from numeric elasticity by 18% on average, which is in accordance with the difference in elongation estimation for the geometric shapes. The analytic method has shown to be multiple orders of magnitude faster than the numerical methods.

**Conclusion:**

It can be concluded that the analytical elasticity computation method has good potential to supplement or replace numerical elasticity simulations in gravity-induced deformations, for shapes that are substantially supported by a planar base perpendicular to the gravitational field. The error is manageable, while the calculation procedure takes less than one second as opposed to multiple minutes with numerical methods. The results will be used in the MRI and Ultrasound Robotic Assisted Biopsy (MURAB) project.

## Introduction

Screening and staging of breast cancer for diagnosis and subsequent treatment is based on medical images acquired on different acquisition modalities and includes mammography (X-ray), ultrasound (US) and MRI.

After image acquisition, proper localization of the tumor is essential for biopsy procedures to take tissue samples or to remove the tumor during surgery. To take full benefit from the previously acquired medical images, the location of the tumor should be aligned from the preoperative imaging into the operating room. The position of the patient can vary from prone during MRI scanning to supine position required for breast surgery for example. During ultrasound scanning and ultrasound-guided biopsy, the patient is returned on her back and additional compression is induced by the ultrasound probe. The computation of the elastic properties will serve as input for real-time adjustments of realistic deformations between preoperative and intra-operative images. For effective deformation models, the elasticity of the model needs to be known with good accuracy, i.e., the difference between computed and actual elasticity must be small. In this study, we aim for a maximum difference in the order of 10%, or at most two times the elasticity variation among FEM-simulated elasticity values. Image registration techniques based on image intensities could be used for small deformations [24], but do not work in cases with large deformations such as the alignment from prone to supine configurations [4].

Deformation of the breast occurs due to body movements. Various physics-based numerical procedures have been presented for biomechanical modeling and soft tissue deformation. The most common computational schemes are based on linear or nonlinear biomechanical models including mass-spring methods (MSM) [2, 7, 20, 23], the mass-tensor method [10, 22], the boundary element method [13, 17] and conventional finite element modeling (FEM) [3, 25, 26].

In an MSM system, an object is modeled by a collection of point masses linked together with massless springs.

Recent studies show the use of FEM to align data with large deformations of the breast [15, 16]. In FEM, a body is subdivided into a set of finite elements (e.g., tetrahedral or hexahedra in 3D, triangles or other polygons in 2D). Displacements and positions of each element are approximated from discrete nodal values using interpolation functions:

1 $$\begin{aligned} \phi (x) = \sum _{i}h_i(x)\phi _i \end{aligned}$$

where $h_i$ is the interpolation function for the element containing *x* and $\phi _i$ is the scalar weight associated with $h_i$. Different choices for the element type and the interpolation functions exist, which depend on the accuracy requirements, geometry of the objects and computational complexity [19]. In general, FEM is used to solve a dynamic problem, which is expressed as partial differential equations (PDEs). These PDEs are then approximated with FEM. The FEM procedure has the advantage that it can handle complicated geometries (and boundaries) of high quality. A dataset of radiological 3D images of the breast anatomy (computed tomography (CT) or MRI) is required to generate a patient-specific FEM. An advantage of MRI is that it shows high sensitivity for detecting breast tumors [8]. The main FEM steps include: tissue classification/segmentation, tissue surface reconstruction, FEM volumetric mesh generation and tissue type assignment for the FEM mesh.

A patient-specific biomechanical model [11] was presented before to provide an initial deformation of the breast before registration between prone and supine MRI images. A zero-gravity reference state for both prone and supine configurations was estimated. The patient-specific unloaded configuration was obtained [12]. The biomechanical methods serve in most cases for the initialization of intensity-based image registration techniques, as in [9] or [18]. The sliding motion of the breast on the chest wall was observed [6], but usually a fixed muscle surface is applied during the FEM simulations [14, 18, 21].

This study introduces a method to analytically derive the elastic modulus of the breast from a pair of MRI scans, taking local differences in tissue density and elasticity into account. The two MRI scans differ by the direction of the gravitational field, which are opposite to each other. Contrary to FEM-based numerical simulations, it is not needed to convert the MRI scan into a volumetric mesh, so mechanical properties on voxel scale are preserved. Also, only one iteration over all voxels is necessary, which makes the method relatively fast

The proposed analytical method requires the breast to be vertically supported by a rigid planar base. As the rib cage is approximately cylindrical, a human breast would need to be supported by a patient-mounted flat plate with a hole for the breast. In an MRI scanner, the breast coil could serve this purpose.

To avoid introduction of significant non-gravity-induced deformations when converting from prone to supine position, it is desirable to use a patient rotation system (PRS) that allows leaving the patient on the bed with breast coil attached, while being flipped over by 180°. Such a system has been developed previously by Whelan et al. [27], which theoretically could be used to take MRI scans of a planar-supported breast in both prone and supine position. It may also be possible to tilt certain MRI scanners such as the 0.25 T G-scan Brio (Esaote SpA, Genoa, Italy), although this is generally limited to rotation over 90°only.

**Fig. 1:**
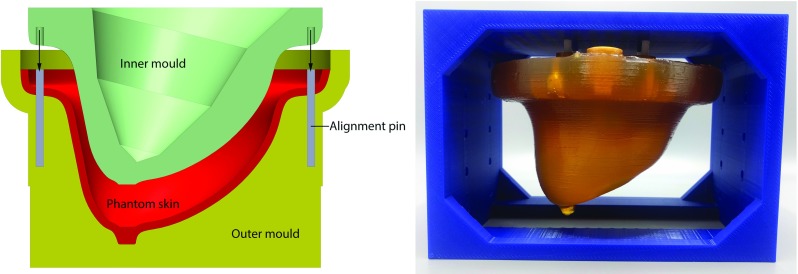
Left: pair of molds (yellow, green) for manufacturing superficial tissue (red). Right: one PVC breast phantom mounted in prone position

## Materials and methods

Four breast phantoms were constructed (Fig. 1, right), consisting of a rigid base with three fiducials, stiff superficial tissue, soft deep tissue and 3–4 lesions.

The superficial and deep tissues and lesions were made of polyvinyl chloride (PVC) with plasticizer mixed in different ratios to obtain different stiffnesses. Contrary to gelatin-based phantoms, PVC is a durable material that can stay intact for extended periods. The superficial tissue consists of relatively stiff PVC which was shaped using a pair of molds (Fig. 1, left) and afterward filled with soft PVC to mimic deep tissue. The lesions were cut in different sizes and shapes from a block of relatively stiff PVC, placed inside the deep tissue at random locations. A rigid frame was put on top and covered with a layer of stiff PVC. The four phantoms which were manufactured this way differ only in the stiffness of deep tissue and the placement of lesions.

**Fig. 2:**
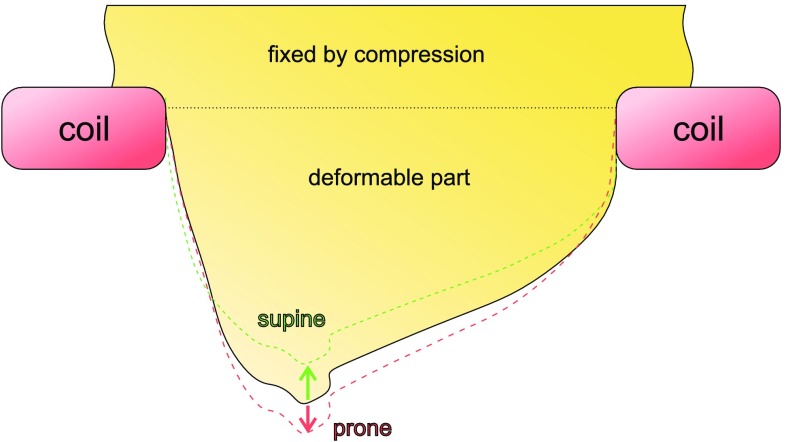
Breast in coil, with gravity-induced deformations in prone and supine positions (dashed lines)

**Fig. 3:**
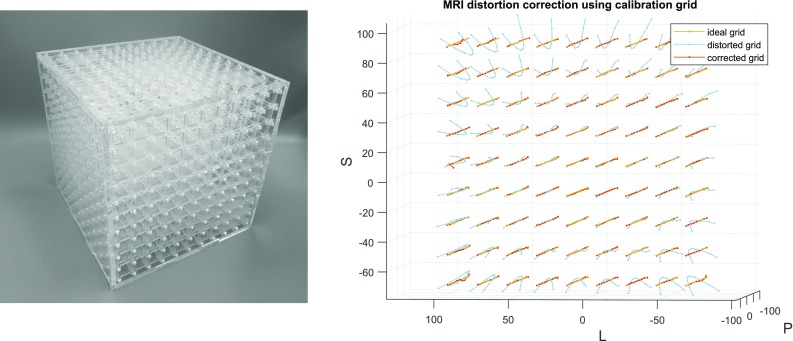
Left: MRI calibration grid. Right: actual (yellow), observed (blue) and distortion-corrected (red) grid locations of the calibration cube

Figure 2 shows the outline of a breast phantom in a neutral reference state. Depending on the orientation (prone or supine), it is deformed by the gravitational field and tip is displaced toward the anterior or posterior direction. The magnitude of these deformations is related to the elasticity, and the approach of the research is to reconstruct the elasticity from these deformations using different methods.

The base represents a rigid inertial frame, which must be planar and normal to the gravitational direction. While a patient’s rib cage provides a rigid supportive base, it is not planar but approximately cylindrical. An external structure such as a breast coil (Fig. 2) may be required to provide this planar support.

Each of the four phantoms was scanned in a 0.25 T MRI scanner (G-Scan Brio) using the 3D balanced steady-state free precession (bSSFP) sequence, with parameters TR = 10 ms, TE = 5 ms, $\hbox {FA}=60^{\circ }$, acquisition resolution $1.5\times 1.8 \times 2.0\, \hbox {mm}$ and isotropic reconstruction resolution $0.94\,\hbox {mm}$.

**Fig. 4:**
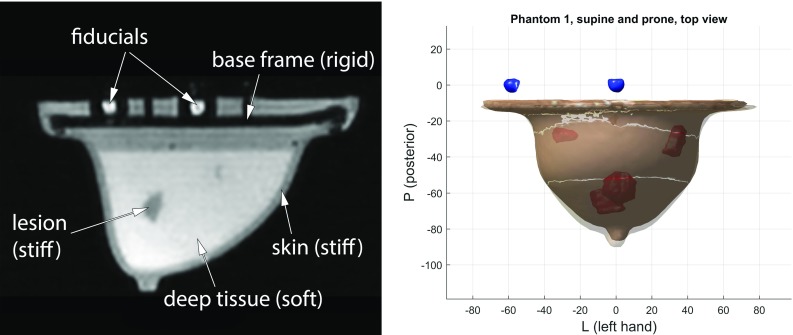
Left: Example sagittal MRI slice. Right: Phantom I in prone and supine configuration, superimposed

The scanner was previously calibrated using a custom 3D calibration grid (Fig. 3, left) from which a fifth-order correction polynomial correction function was constructed. The ideal, distorted and corrected grid patterns are shown in Fig. 3. The measured residual error is $0.2\, \hbox {mm}$, so sub-pixel resolution is feasible.

The distortion-corrected MRI scans (Fig. 4, left) were segmented by intensity thresholding and automatically aligned with a rigid transformation using the three fiducials, in which the root-mean-square registration error was found to be 0.2–0.3 mm. From these data, surface and volumetric meshes in different levels of detail were constructed.

Figure 4 right shows two configurations of phantom I, overlaid on each other, after segmentation and registration. A significant displacement of the tip resulting from the change in gravity field direction can be observed.

## Elasticity estimation

### Preamble

The deformation of an object in a gravitational field is the result of elongations of tissue, which depends on the local ratio of tensile stress $\sigma$ and Young’s modulus *E*:

$$\begin{aligned} \epsilon = \frac{\sigma }{E} \end{aligned}$$

The stress at a given location is primarily induced by the weight of the masses below that location, and also influenced by interactions with surrounding tissue. In the general case, the resulting stress distribution in the object is a complex pattern and cannot be solved analytically, requiring simulations to quantify the deformations. However, in our case, we can use the knowledge that the object’s attachment to the rigid frame is planar and perpendicular to the gravity direction, when in prone and supine positions. For objects with a constant cross section such as a block or a cylinder, it can be shown (see “Analytical derivation of elasticity” section) that the deformation displacement can be solved analytically.

We introduce the assumption that the tensile stress $\sigma$ solely depends on the vertical position in the object, i.e., it is constant within any planar cross section parallel to the base. It can be shown that this assumption is valid for blocks, cylinders and prism-shaped objects which have a constant cross section. For the breast phantom shapes, the assumption can be justified by the fact that the masses of the whole breast are substantially positioned below the rigid base. To validate this assumption, the stress distribution and elongation for a range of geometric shapes are also investigated.

**Fig. 5:**
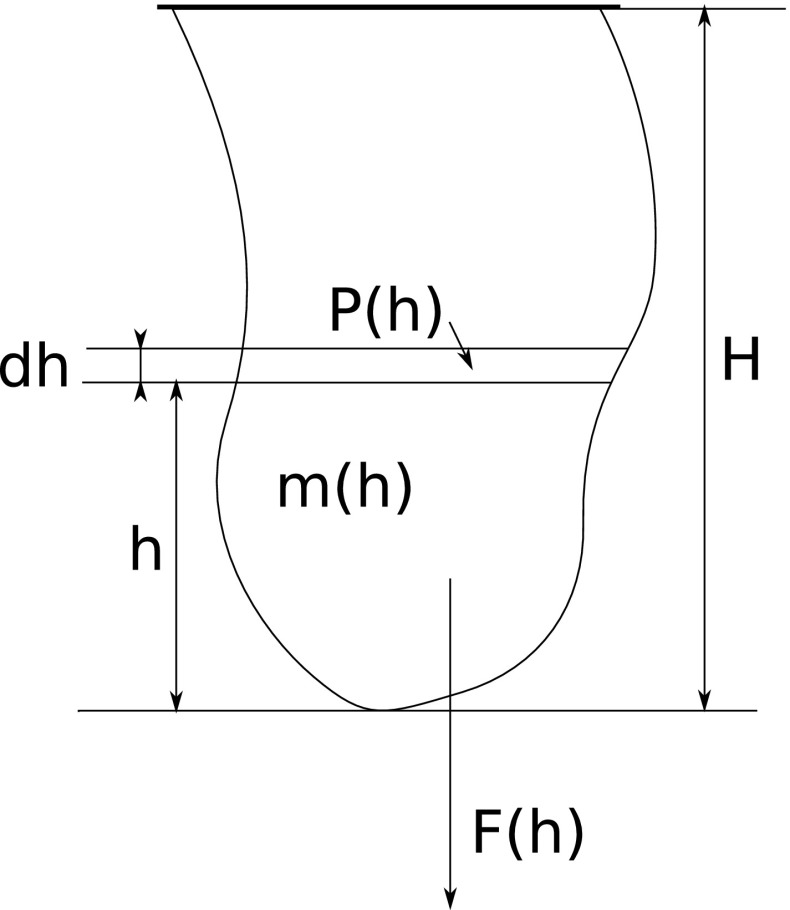
Schematic view of force and pressure at a given height

### Analytical derivation of elasticity

Figure 5 schematically shows the forces and pressures acting on a shape with inhomogeneous density and elasticity, hanging from a planar, rigid attachment on the top. At a given height *h*, the cross-sectional area is *A*(*h*), the mass of the body below it is denoted as *m*(*h*) and the gravitational force acting on it *F*(*h*). We now derive expressions for the vertical stress $\sigma (h)$ and elongation $\epsilon (h)$ for every height, leading to a formula for the displacement *D* of the lower extremity of the body.

The total mass of the body up to height *h* is given as:

2 $$\begin{aligned} m(h) = \int ^h_0 \iint \rho (x,y,z)\mathrm{d}x\mathrm{d}y\mathrm{d}z \end{aligned}$$

The gravitational force acting on the slice at height *h* is calculated as:

3 $$\begin{aligned} F(h) = m(h) g \end{aligned}$$

The tensile stress in the slice is generally not constant, and its exact distribution depends on many levels of tissue interactions. We are interested in the mean tensile stress $\overline{\sigma }(h)$, which is found by dividing the gravitational force by the slice’s cross-sectional area:

4 $$\begin{aligned} \overline{\sigma }(h) = \frac{F(h)}{A(h)} = \frac{m(h)g}{A(h)} \end{aligned}$$

The tissue elasticity is also inhomogeneous in general, with local Young’s modulus $E(\mathbf {r})$, again averaged to $\overline{E}(h)$ for height *h*. The local relative elongation is $\epsilon = \Delta L/L_0 = \sigma (\mathbf {r})/E(\mathbf {r})$, and the mean elongation at height *h* is given as:

5 $$\begin{aligned} \overline{\epsilon (h)} = \frac{\overline{\sigma }(h)}{\overline{E}(h)} = \frac{m(h)g}{A(h)\overline{E}(h)} \end{aligned}$$

The total displacement of the body’s lower extremity is found by integrating all infinitesimal elongations:

6 $$\begin{aligned} D = \int _{0}^{H} \overline{\epsilon }(h) \mathrm{d}h = g \int _{0}^{H} \frac{m(h)}{A(h)\overline{E}(h)}\mathrm{d}h \end{aligned}$$

The purpose of this study is to find the average Young’s modulus *E* from a pair of gravity-induced body displacements. To preserve differences in (mean) elasticity among slices, we factorize every slice’s elasticity into a constant factor *E* and a layer-specific adjustment factor $\hat{E}(h)$:

7 $$\begin{aligned} \overline{E}(h) = E \hat{E}(h) \end{aligned}$$

The displacement equation can now be written as follows:

8 $$\begin{aligned} D = \frac{g}{E} \int _{0}^{H} \frac{m(h)}{A(h)\hat{E}(h)}\mathrm{d}h \end{aligned}$$

It can split into an object-specific intrinsic part which remains constant across all simulations, and an extrinsic (variable) part depending on *g* and *E* only. The intrinsic part $\beta$ is defined as:

9 $$\begin{aligned} \beta = \int _{0}^{H} \frac{m(h)}{A(h)\hat{E}(h)}\mathrm{d}h \end{aligned}$$

Substituting into *D* gives:

10 $$\begin{aligned} D = \beta \frac{g}{E} \end{aligned}$$

For the scanned breast phantoms, we, therefore, assume that the displacement (for small displacements) is linear in *g* / *E*, with proportionality factor $\beta$. The $\beta$ value can be estimated from DICOM data, in combination with knowledge of the materials. For PVC phantoms, its density was measured to be $\rho = 1.075\,\hbox {g} / \hbox {cm}^{3}$.

Analyzing the prone and supine scans of a phantom, we have $\beta _\mathrm{p}$ and $\beta _\mathrm{s}$ for prone and supine, respectively. In general, $\beta _\mathrm{p} \ne \beta _\mathrm{s}$, because the shapes are significantly different: The total volume and cross-sectional area at the base are approximately equal, but due to difference in height the cross-sectional shape is more squeezed in prone position than in the supine one.

The phantom height *H* is ill-defined due to possible irregularities at the tip, but the difference $\Delta H = H_\mathrm{p}-H_\mathrm{s}$ can be accurately determined by comparing point clouds around the tip using, e.g., the iterative closest point algorithm [5], and optimizing $\Delta H$ such that the total point distance is minimal, or alternatively by comparing the centroids of the point clouds.

The parameter we want to compute is the Young’s modulus *E*. When no forces act on the phantom, it would have some shape halfway the prone and supine shapes. The tip displacement to either prone or supine shape in a gravitational field *g*, is $\Delta H/2$. We can now derive the Young’s modulus *E* as follows:

11 $$\begin{aligned} \beta _n= & {} \frac{\beta _\mathrm{p}+\beta _\mathrm{s}}{2} \end{aligned}$$

12 $$\begin{aligned} E= & {} \frac{\beta _n g}{\Delta H/2} = \frac{2 (\beta _\mathrm{p}+\beta _\mathrm{s}) g}{\Delta H} \end{aligned}$$

### Numerical simulation of deformations

The purpose of FEM simulations is to determine the elasticity *E* of the different phantoms, based on the segmented models. The general strategy is to apply a gravitational field to the FEM model of a phantom in a specific direction. This deformed model is then compared to a reference phantom which was scanned in a different orientation, providing information about the elasticity parameter.

In the following subsections, we present two strategies to find the Young’s modulus by simulation, of which one strategy is performed by two different simulation software packages.

#### Estimating the $\beta$ values by simulation in SOFA

In “Analytical derivation of elasticity” section, we have introduced a method to derive the values of $\beta$ for the four phantoms in different orientations directly from a DICOM scan. In this section, we find $\beta$ by simulation in SOFA at five different mesh resolutions [1]. For each mesh resolution, we have run a simulation with the phantom’s Young’s modulus set to $E=6000\,\hbox {Pa}$ and gravity $g=2.0\, \hbox {m/s}^{2}$. After 100 iterations, the simulation has stabilized and the vertices of the mesh in this configuration were extracted and analyzed. The displacement from the initial position follows by comparing the point clouds around the tip. The value of $\beta$ then follows from Eq. (10). This procedure is repeated for each resolution of the mesh and for both prone and supine orientations, then the mean $\beta _\mathrm{s}$ and $\beta _\mathrm{p}$ values were computed. From the $\beta _\mathrm{s}$, $\beta _\mathrm{p}$ and $\Delta H$, and assuming linearity of the displacement to *g* / *E* ratio, the Young’s modulus *E* can be derived using Eqs. (11) and (12).

#### Supine–prone and prone–supine simulation and matching in SOFA and Febio

Taking a phantom scanned in supine configuration, the base of the phantom is immobilized and a force field sized two times the gravity ($19.62\,\hbox {m/s}^{2}$) in anterior direction is applied to the phantom. After stabilization in simulation, the final state is extracted and compared to the phantom in prone position, which serves as the reference phantom.

The error value, $\epsilon$, is defined as the distance between the simulated and reference phantoms in the area around the tip of the breast and can be positive or negative. The actual value is dependent on the elasticity parameter *E* of the phantom, which is optimized to bring $\epsilon$ to zero.

The minimization is performed using the Newton’s method computed over E and the distance error, corrected by an adaptive step approach (when the FEM analysis software diverges). When procedure ends, i.e., when the method achieves a pre-defined error or when it reaches a maximum number of iterations, the estimated E parameter is returned with its associated error.

The procedure is then repeated for the opposite direction (prone to supine). In general, this also leads to a different *E* value. The mean value (square-harmonic mean-root) of $E_\mathrm{sp}$ and $E_\mathrm{ps}$ is then taken as the elasticity of the final phantom.

**Fig. 6:**
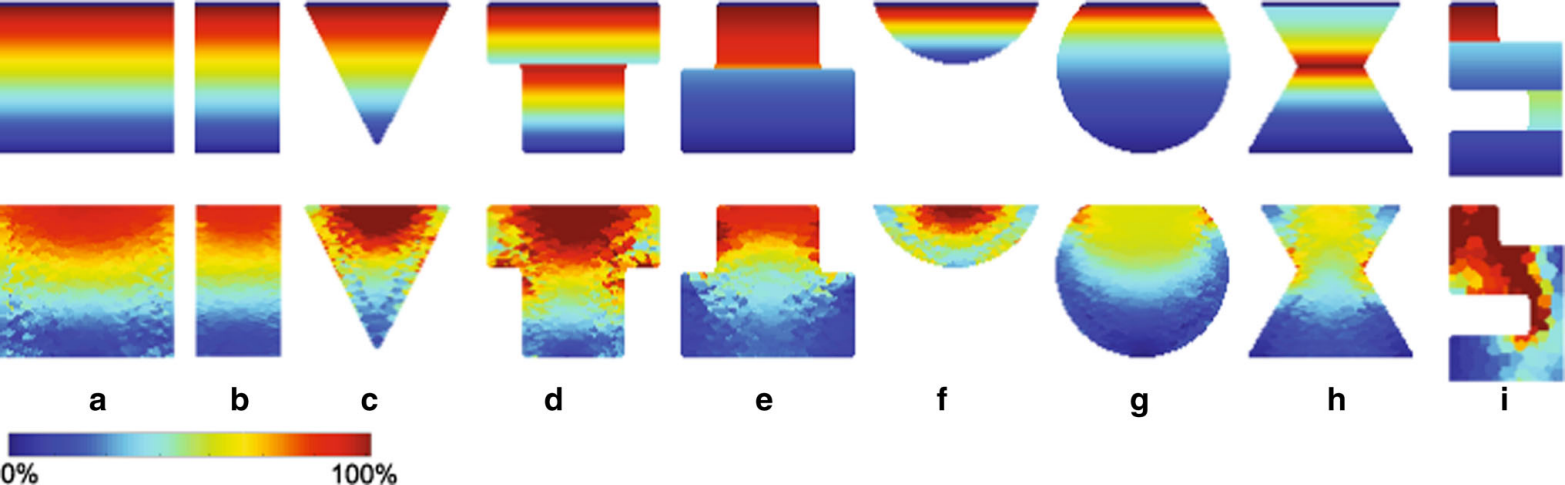
Analytically derived tensile stress (top row) compared with simulated stress (bottom row) for a selection of geometric shapes

## Results

### Validation of analytical stress calculation on geometric shapes

Nine homogeneous geometric shapes were generated and analyzed: two cylinders with different aspect ratios, a cone, a T-piece in normal and upside-down orientation, a half sphere, a sphere, an hourglass and a snake-like shape.

Figure 6 shows the stress distribution along the vertical midway plane for all nine shapes. The first row uses the analytical computation method. The assumption that the stress distribution is constant in a cross-sectional area parallel to the base, is reflected in having constant colors in horizontal direction. The second row shows the tensile stress from numerical simulations using the SOFA software package under the same conditions.

Table 1 lists the calculated and simulated $\beta$ values for the same geometric shapes.

The following observations can be made:

- For cylinder, cubic and prism-like shapes that have a constant cross-sectional area (a and b), the numerically derived stress distribution matches the analytically derived one quite well. The $\beta$ values derived by both methods are well comparable (deviation under 9%).
- For shapes that do not have a constant cross-sectional area, but are substantially vertically supportive (c-h), the analytically calculated and SOFA-simulated $\beta$ values are still comparable (deviation up to 18%) although the stress distribution is different.
- For shapes in which the lower extremity is not vertically supported by the base, i.e., no vertical line of maximum height can be drawn that entirely lies within the model (i), both the analytically calculated $\beta$ value and the stress distribution are inconsistent with simulations.

**Table 1:** Calculated and simulated $\beta$ values for the nine geometric shapes

Geometric shape	Calculated $\beta$	Simulated $\beta$
a	2375	2169
b	2373	2229
c	772	724
d	1638	1581
e	4500	4979
f	213	205
g	1932	2276
h	3802	3797
i	4942	26,499

### Analytical derivation of elasticity of phantoms

Each of the four phantoms was scanned in prone and supine position, and from the resulting DICOM scans, the $\beta _\mathrm{p}$ and $\beta _\mathrm{s}$ values are computed using Eq. 9 and assuming a homogeneous density and elasticity distribution. From these values plus the observed vertical displacements, the *E* parameters are computed using Eq. 12 and the results are listed in Table 2. It can be observed that phantom IV has the highest $\beta$ and *E* values, making it the stiffest phantom, while phantom II is the softest one. In general, the $\beta$ values are higher in prone position, which is as expected.

### Simulation of $\beta$ in SOFA

For numerical FEM simulations, each DICOM scan was segmented and meshed at five different levels of detail and subsequently simulated in the SOFA simulation package. The resulting $\beta$ values of the four phantoms (in both orientations) plus the averaged *E* value are listed in Table 3. Calculation of each $\beta$ value requires ten simulation runs in SOFA, lasting a few minutes in total.

**Table 2:** Analytically derived properties of four phantoms, under the assumption of constant tensile stress in each cross section

Phantom	$\beta _\mathrm{s}$	$\beta _\mathrm{p}$	$\Delta H$	*E*
I	1215	1298	3.28	7514
II	1129	1269	4.73	4972
III	1356	1444	3.58	7673
IV	1420	1471	2.93	9677

**Table 3:** Properties of four phantoms, derived by numerical simulation in SOFA in five different resolution scales and then averaged

Phantom	$\beta _\mathrm{s}$	$\beta _\mathrm{p}$	$\Delta H$	*E*
I	$1007 \pm 58$	$1134 \pm 38$	3.28	$6403 \pm 207$
II	$947 \pm 41$	$1125 \pm 36$	4.73	$4297 \pm 113$
III	$1131 \pm 48$	$1259 \pm 61$	3.58	$6549 \pm 213$
IV	$1170 \pm 43$	$1383 \pm 34$	2.93	$8548 \pm 184$

**Fig. 7:**
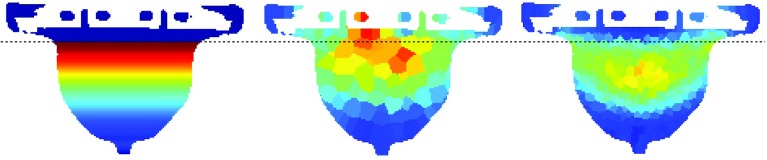
Tensile stress for phantom I in the transversal plane, in supine position. Left: derived using analytical method. Center and right: numerically simulated using SOFA in low resolution (center) and high resolution (right). The dashed line indicates the boundary plane between the rigid and deformable parts

Figure 7 shows the analytically derived stress distribution in the transversal plane of phantom I in supine configuration together with the numerically simulated stress distribution in the same plane at low and high resolutions. It can be observed that the resulting stress patterns are comparable to that of certain geometric shapes in Fig. 6a–h. Only the analytic method shows a sharp transition at the boundary layer, as the analytical method uses slices with thickness of one voxel while the FEM-based method subdivides the volume in a different way.

### Numerical simulation by supine–prone and prone–supine matching in SOFA

Table 4 lists the elasticities obtained by numerical simulation from supine to prone position and vice versa, in SOFA. As opposed to the $\beta$ computation method, the prone–supine simulation method also takes nonlinearities into account which theoretically results in a more accurate estimate of the *E* value.

For each resolution, up to ten simulation runs are needed to find the final *E* value in which the error vanishes. This makes the method relatively slow, requiring about twenty minutes of computation time on a quad-core 2.5 GHz computer per phantom. By parallelizing computations of the four phantoms, the total computation time for all *E* values was measured to be approximately half an hour.

**Table 4:** Elasticity values found by numerical simulations from supine-to-prone ($E_\mathrm{sp}$) and prone-to-supine ($E_\mathrm{ps}$) in four different resolution scales and then averaged, using SOFA

Phantom	$E_\mathrm{sp}$	$E_\mathrm{ps}$	Mean *E*
I	$5047 \pm 374$	$6459 \pm 373$	$5688 \pm 272$
II	$3395 \pm 189$	$4513 \pm 272$	$3895 \pm 159$
III	$5381 \pm 376$	$6828 \pm 438$	$6040 \pm 288$
IV	$6245 \pm 433$	$7445 \pm 322$	$6805 \pm 283$

**Table 5:** Elasticity values found by simulating from supine-to-prone ($E_\mathrm{sp}$) and prone-to-supine ($E_\mathrm{ps}$) in four different resolution scales and then averaged, using FEBio as software package

Phantom	$E_\mathrm{sp}$	$E_\mathrm{ps}$	Mean *E*
I	$5046 \pm 272$	$5252 \pm 307$	$5145 \pm 254$
II	$4290 \pm 351$	$4298 \pm 273$	$4291 \pm 276$
III	$5291.52 \pm 383$	$5639 \pm 456$	$5459 \pm 400$
IV	$7916 \pm 1165$	$7564 \pm 957$	$7731 \pm 1016$

### Numerical simulation by supine–prone and prone–supine matching in FEBio

Table 5 lists the elasticity values using the FEBio software package. The resulting elasticity values are comparable to those obtained by SOFA. A relatively high variance is present in phantom IV, which may be caused by side effects in the software package.

**Fig. 8:**
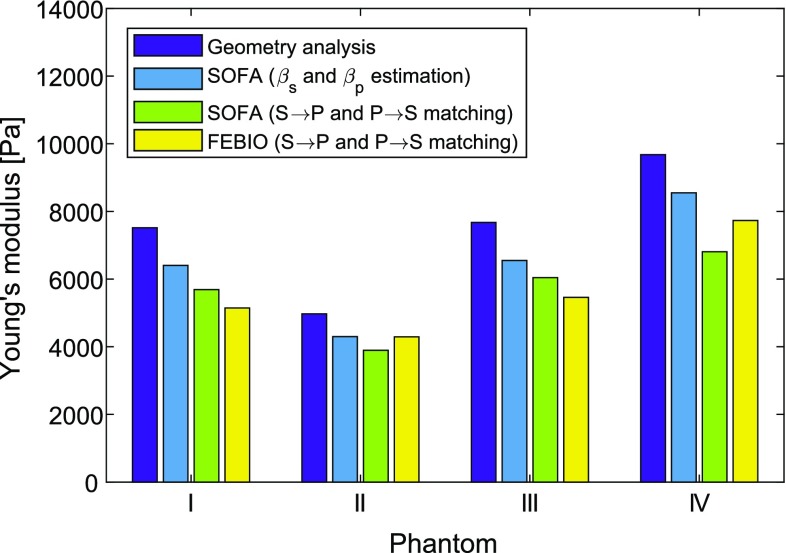
Young’s modulus for four phantoms, derived by four different methods

### Comparison of different elasticity measurement methods

Figure 8 graphically shows the elasticity of the four phantoms, derived using the different methods, while Table 6 lists the overall phantom elasticities, averaged from the four different methods.

Two methods using SOFA were presented: The first one numerically simulates the $\beta _\mathrm{s}$ and $\beta _\mathrm{p}$ values from the supine and prone meshes separately and measures the tip displacement *D*, from which the phantom’s elasticity *E* is derived. The second method involves finding *E* directly by simulation from supine to prone position such that the tip position error is eliminated. The first method seems to give consistently higher estimates for *E*, especially for phantom IV. Possible causes might be the nonlinearity of the displacement-to-*g* / *E* ratio, i.e., $\beta$ cannot be considered constant for the required range of displacements. Furthermore, the deformations of the tip resulting from proper FEM simulations influence the displacement calculations. As the second algorithm uses the iterative point cloud algorithm to minimize tip displacements and also takes nonlinear effects into account, that one can be considered more accurate than the first one.

**Table 6:** Mean elasticity values for each phantom, taken as the average of the separate values derived by the four different methods

Phantom	Mean *E*
I	$6188 \pm 886$
II	$4364 \pm 387$
III	$6430 \pm 815$
IV	$8190 \pm 1057$

The numerical results from FEBio simulations are in accordance with SOFA matching simulations, which is an indication that the simulations are consistent.

## Discussion

We have presented a new method to analytically evaluate the elasticity of breast phantoms, from a pair of MRI scans in prone and supine position. The values found from analyzing the gravity-induced deformations are comparable to the elasticities derived from FEM simulations using FEBio and SOFA, with deviations of up to 18%. A study on nine geometric shapes has shown that the method is not only applicable to breast shapes, but also to other bodies and geometric objects as long as it is substantially supported by a planar rigid base.

The advantages of the analytical method are that the elasticity calculation is very fast ($< 1$ s) and takes each individually scanned voxel into account, without need for mesh generation. As the voxel intensity in a scan gives certain information about tissue type, density and/or elasticity (depending on scanning protocol), tissue inhomogeneities can be directly incorporated in the analytical computations. The main limitations are that the method is only suitable for deformations in the linear range, and that the shapes must be substantially supported by a planar base perpendicular to the gravitational field.

The fact that a human breast is relatively flexible and the chest wall is not planar but cylindrically shaped, makes clinical application difficult. An artificial planar support base could be constructed by using a patient-mounted breast coil, ideally in combination with a patient rotation system. The presented methods may also have applications in different domains, wherever deformation of bodies is involved in situations that meet the aforementioned boundary conditions The conclusion is that under specific conditions, the elasticity of a deformable object such as a human breast can be quickly computed from a pair of volumetric scans with sufficient accuracy, without need for FEM simulations. This promising result opens the door to new applications which can benefit from this complementary and near-real-time elasticity computation method.
